# Evolution of ordered nanoporous phases during h-BN growth: controlling the route from gas-phase precursor to 2D material by *in situ* monitoring†

**DOI:** 10.1039/d2nh00353h

**Published:** 2022-09-21

**Authors:** Adrian Ruckhofer, Marco Sacchi, Anthony Payne, Andrew P. Jardine, Wolfgang E. Ernst, Nadav Avidor, Anton Tamtögl

**Affiliations:** Institute of Experimental Physics, Graz University of Technology Graz Austria tamtoegl@tugraz.at; Department of Chemistry, University of Surrey Guildford GU2 7XH UK; Cavendish Laboratory J. J. Thompson Avenue Cambridge CB3 0HE UK na364@cantab.ac.uk

## Abstract

Large-area single-crystal monolayers of two-dimensional (2D) materials such as graphene and hexagonal boron nitride (h-BN) can be grown by chemical vapour deposition (CVD). However, the high temperatures and fast timescales at which the conversion from a gas-phase precursor to the 2D material appears, make it extremely challenging to simultaneously follow the atomic arrangements. We utilise helium atom scattering to discover and control the growth of novel 2D h-BN nanoporous phases during the CVD process. We find that prior to the formation of h-BN from the gas-phase precursor, a metastable (3 × 3) structure is formed, and that excess deposition on the resulting 2D h-BN leads to the emergence of a (3 × 4) structure. We illustrate that these nanoporous structures are produced by partial dehydrogenation and polymerisation of the borazine precursor upon adsorption. These steps are largely unexplored during the synthesis of 2D materials and we unveil the rich phases during CVD growth. Our results provide significant foundations for 2D materials engineering in CVD, by adjusting or carefully controlling the growth conditions and thus exploiting these intermediate structures for the synthesis of covalent self-assembled 2D networks.

## Abstract 2

Diffraction measurements following the growth of h-BN, reveal intermediate long-range ordered open network structures during the formation. DFT calculations explain their nature, outlining new routes to control the synthesis of covalent 2D networks.

## Graphical abstract

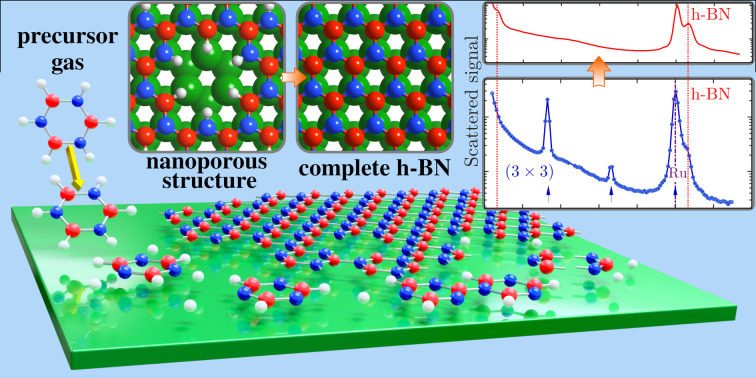

**New concepts**

As a key to understand both the formation and stability of 2D materials grown *via* on-surface synthesis, the individual steps, transformations and multiple pathways from the gas-phase precursor to the 2D layer need to be controlled and understood. However, the temporal evolution of these steps has so far only briefly been discussed in the context of graphene formation and no experimental reports on the individual steps have been reported up to now. We demonstrate that precise control of the growth of 2D materials is achievable by employing *in situ* time-resolved He atom scattering, which reveals products formed by the decomposition of the gas-phase precursor on the metal substrate. We exploit the technique to observe two previously unreported 2D phases of hexagonal boron nitride constituted by open-porous networks. Our work shows that it is possible to directly monitor the growth kinetics of 2D materials and isolate intermediate and metastable phases, paving the way to a greater control of 2D materials by chemical vapour deposition.

## Introduction

**Fig. 1:**
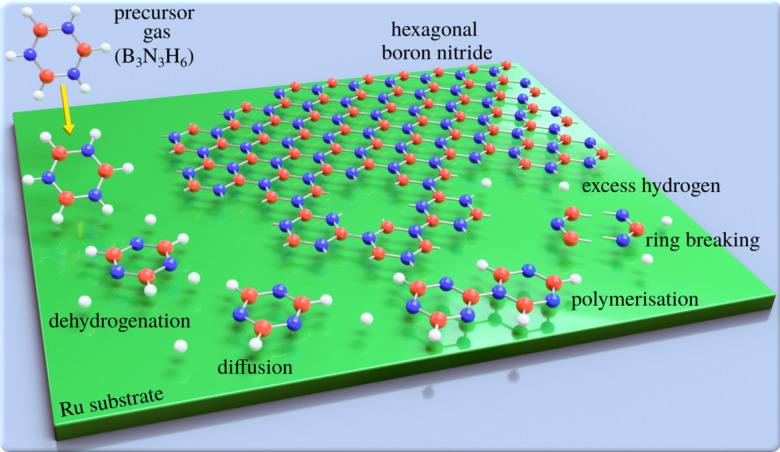
Schematic illustrating the epitaxial growth of h-BN by chemical vapour deposition: a gaseous precursor (*e.g.* borazine, B_3_N_3_H_6_) is brought into contact with a (hot) catalyst surface (Ru), triggering the chemical reactions such as breaking of the borazine rings and dehydrogenation, followed by the assembly of the epitaxial overlayer.

Two-dimensional (2D) materials such as graphene and hexagonal boron nitride (h-BN) offer technological promise,^1,2^*e.g.* with h-BN being considered to be the “ideal” dielectric for 2D based field-effect transistors.^3^ However, their properties are highly dependent on the perfection of the 2D layers. For this reason, intense efforts have been devoted to study and improve the growth of defect-free 2D materials.^4,5^ A promising method of synthesising large-area 2D layers is chemical vapour deposition (CVD) and the CVD synthesis of atomically thin h-BN on metal substrates is described in several review articles.^6,7^ The process, which is illustrated in Fig. 1, involves a gas-phase precursor deposited on a solid substrate at elevated temperatures. By diffusion^8^ and dehydrogenation or fragmentation of the precursor, the adsorbates are attached to growing clusters and eventually form the 2D layer. A complete dehydrogenation of the precursor requires overcoming multiple energy barriers. As a result, it might be expected that at intermediate temperatures, dehydrogenation would not be complete, which in-turn can result in metastable or intermediate structures. For the synthesis of bulk h-BN it is known that the process involves several steps of borazine-polymerisation.^9–12^ There are several routes, but even in the bulk, the process has not been studied in great detail. Here, we follow a series of structural changes to identify intermediate structures in 2D growth.

While it is crucial to understand the growth process, mechanistic and kinetic studies are rare and mostly focus on the growth of nanocarbons.^13–16^ Dehydrogenation and intermediate structures during CVD of 2D materials have been proposed,^17–23^ but to the best of our knowledge have not been studied experimentally. In general, kinetics and the thermochemistry of intermediate products, may lead to metastable structures. However, phase-diagrams due to partially dehydrogenated precursors have not been reported. Most studies report completed overlayer structures while the complexity and individual steps, as illustrated in Fig. 1, are often ignored. In particular, previous h-BN studies using real space methods^24–27^ concentrate on local order in completed h-BN structures, while reciprocal space studies^28–30^ have provided information about long-range order.^31^

In this paper we present a systematic analysis, at various temperatures beyond the ones reported for best growth conditions (1050 to 1100 K^24,26,29^) and at various dosing rates. By following h-BN growth *in situ* using helium atom scattering (HAS) we demonstrate the existence of metastable structures during the formation of h-BN from borazine (B_3_N_3_H_6_). HAS is a well-established technique for monitoring thin-film growth modes^32–34^ and has been used to study the quality of CVD-grown 2D materials^35–38^ and inter-layer interaction,^39^ yet investigations of intermediate structures have not been performed. In particular, we find that there is one precursor structure with a well-defined (3 × 3) periodicity, meaning a well-defined route for the polymerisation reaction which leads to h-BN. We further find that by dosing excess borazine, a (3 × 4) structure forms, which could be attributed to a partially polymerised second-layer on top of the formed h-BN.

Our experimental results are complemented by van der Waals (vdW) corrected density functional theory (DFT) calculations which confirm the nature of the system, helping us to determine which self-assembled structures are compatible with the experimental results.

## Results and discussion

The adsorption of the precursor gas (borazine, B_3_N_3_H_6_) on the Ru substrate has been investigated in several other studies using Auger electron spectroscopy, X-ray photoelectron spectroscopy, electron energy loss spectroscopy and low energy electron diffraction.^40–42^ There is general consensus in the literature, that borazine only adsorbs molecularly at low (<140 K) temperatures^41–43^ with dehydrogenation setting in at temperatures of 150–250 K, depending on the substrate.^41,42,44^ Starting from about 600 K, depending on the metallic substrate, the B–N ring is reported to break down into its atomic constituents.^43,44^ According to Paffet *et al.* 1000 K are necessary for h-BN formation on Ru(0001)^40,41^ while hydrogen desorption occurs over a wide temperature range^42^ and may even intercalate below the h-BN layer.^45,46^

Helium diffraction allows *in situ* measurements even at growth temperature, and is known for its unique sensitivity to adsorbates, including hydrogen atoms.^47–55^ Furthermore, unlike other established techniques,^56^ HAS is completely inert and does not modify the process under investigation.^57^ While the specular reflection gives an estimate of adsorbate coverage on the clean surface, the angular distribution provides insight in the time evolution of periodic structures being formed on the surface.^31,36^ In the present work, CVD growth was performed at a set crystal temperature while monitoring the surface using repeated one-dimensional angular diffraction scans, where we observe the emergence and disappearance of additional superstructures followed by the formation of h-BN.

All experimental data was obtained with the Cambridge spin-echo apparatus which uses a nearly monochromatic atomic beam of ^3^He, scattered off the sample in a total scattering angle of 44.4° and with an incident energy of 8 meV (see Experimental section in the ESI†). The parallel momentum transfer Δ*K* is given by Δ*K* = |Δ**K**| = |**K**_f_ − **K**_i_| = |**k**_i_|(sin *ϑ*_f_ − sin *ϑ*_i_), with **k**_i_ being the incident wavevector and *ϑ*_i_ and *ϑ*_f_ the incident and final angles with respect to the surface normal, respectively.^58–60^ Compared to techniques such as scanning tunnelling microscopy (STM), HAS averages over larger surface areas, typically ≈3 mm^2^. Therefore, the advantage of HAS is to give precise information about any long-range periodicity of surface structures. For ease of comparison, the diffraction scans for the different structures are plotted as a function of the parallel momentum transfer, Δ*K*, relative to the G_01_ peak of the Ru(0001) substrate, |Δ*K*/*G*_01_|. By converting the abscissa in this way, the position of the observed diffraction peaks directly relates their periodicity with respect to the substrate lattice spacing.

After formation of the complete 2D layer, HAS can also be used to determine the lattice constant, crystal quality and bonding to the substrate of the 2D material as *e.g.* shown for CVD-grown graphene.^37,39,61,62^ As the main focus of this work is the route from the gas-phase precursor to h-BN, the periodicity, reconstruction and quality of the complete h-BN layer is instead discussed in Section 5 of the ESI.†

### A precursor structure to h-BN growth

First, we describe how borazine exposure at low temperature (*T* < 880 K) reveals a precursor structure on Ru(0001), which by further annealing at *T* = 880 K can be converted to h-BN.

**Fig. 2:**
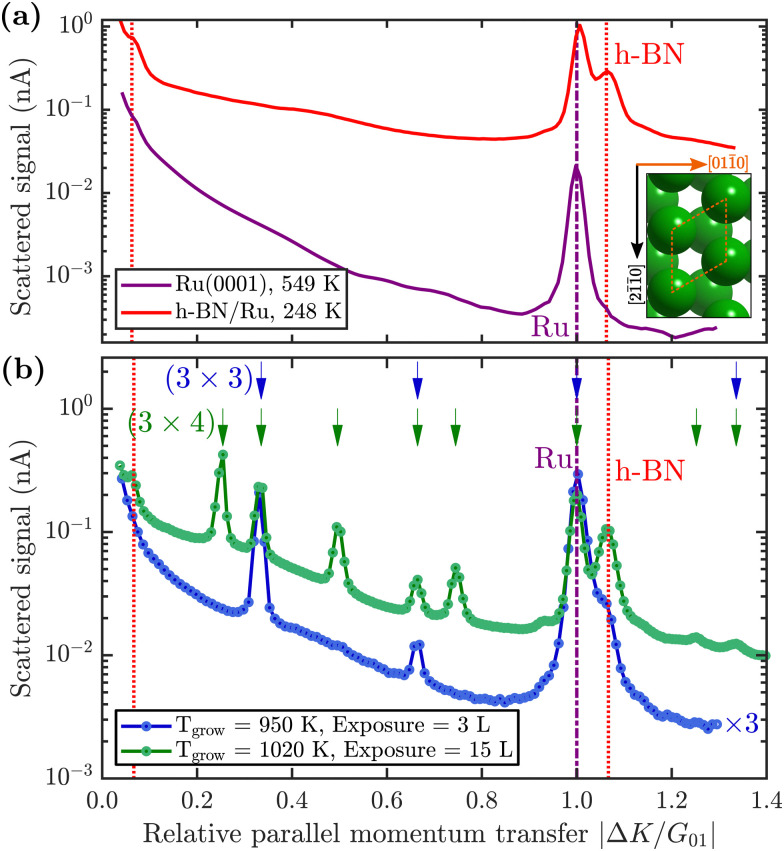
(a) Comparison of the angular diffraction scans for clean Ru(0001) in purple and the completed h-BN overlayer on Ru in red. The [011̄0] scanning direction (
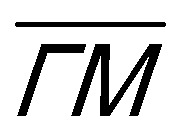
) is shown in the inset. The purple dash-dotted line indicates the position of the first order Ru diffraction peak and the red dotted lines the h-BN Moiré peaks. (b) Diffraction scans during borazine exposure reveal additional superstructures, depending on the growth temperature and total exposure. The position (shown by the arrows at the top) and spacing of the additional peaks reveal a (3 × 3)/(3 × 4) superstructure plotted in blue/green. Low exposure at lower temperatures reveals a (3 × 3) structure (blue curve, grown at 950 K), while at higher exposures and higher temperatures an intermediate (3 × 4) pattern emerges (green curve, grown at 1020 K). To improve the signal to noise ratio, the sample was subsequently cooled down for the duration of both scans and the blue curve was scaled by a factor of 3 to facilitate comparison.

The purple line in Fig. 2(a) shows a diffraction scan of the clean Ru(0001) substrate. Exposing Ru to 7 Langmuir (L) of borazine at a surface temperature of 600 K, results in decreased diffraction intensities and helium reflectivity. Moreover, the lack of any additional diffraction peaks is typical of a disordered structure.^55^ The behaviour is consistent with earlier studies showing that the B–N ring starts to break down into its constituents only above about 600 K.^40,43,44^

Upon increasing the temperature to 750 K while maintaining borazine overpressure, additional peaks start to appear between the specular and first order Ru diffraction peaks. Fig. 2(b) (blue curve) shows the characteristic diffraction pattern that emerges. Equidistant peaks at |Δ*K*/*G*_01_| = 0.33 and 0.66 indicate a (3 × 3) periodic structure on the surface, which we label BN_I_. If dosing is performed at even higher temperatures (*T* ≥ 880 K), in addition to the observed BN_I_ structure, a shoulder appears to the right-hand side of the first order Ru diffraction peak, indicating the formation of a h-BN structure on the surface (vertical red dotted line in Fig. 2). The peak, which occurs at |Δ*K*/*G*_01_| = 1.08, is a result of the commensurate Moiré pattern on Ru^29^ (see also h-BN periodicity in the ESI†).

**Fig. 3:**
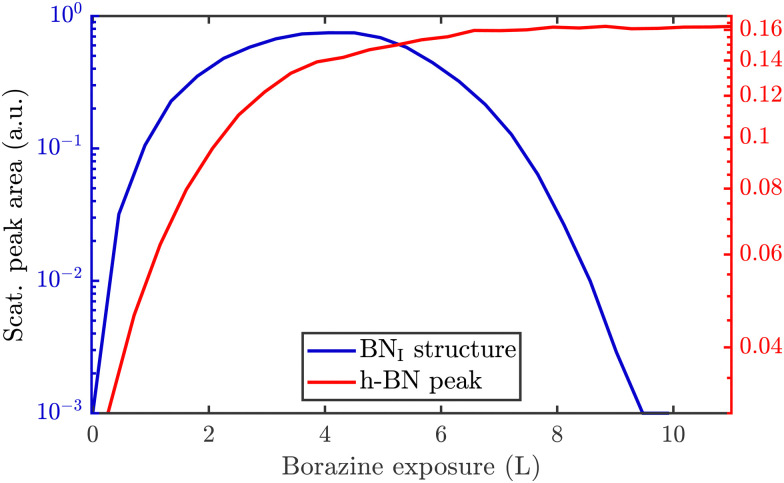
*In situ* monitoring of the integrated peak intensities reveals that the BN_I_ structure acts as precursor to the h-BN overlayer. The characteristic diffraction peaks for the BN_I_ structure and the h-BN peak are plotted *versus* borazine exposure at a substrate temperature of 880 K. The BN_I_ structure increases prior to the h-BN intensity and has already disappeared when the h-BN intensity exhibits its maximum.

Since a (3 × 3) superstructure composed of intact borazine molecules shows only weak binding to the substrate, the observed BN_I_ structure, as shown later in our DFT calculations, must be composed of partly dehydrogenated borazine molecules, in line with the reported low experimental dehydrogenation temperature on other substrates.^44^Fig. 3 illustrates *in situ* monitoring of the integrated peak intensities, which demonstrates that the BN_I_ structure precedes the growth of h-BN. The exposure dependent intensities are obtained from repeated angular diffraction scans. Immediately after dosing begins, the (3 × 3) peaks start to rise rapidly (blue line), while only after a short delay the h-BN diffraction peak increases (red line), although less quickly than the BN_I_ structure. The h-BN peak intensity reaches its maximum at the same point where the BN_I_ structure disappears. We conclude that the BN_I_ structure is converted into h-BN and acts as a precursor structure to the complete h-BN overlayer. Since the intensity of the BN_I_ structure drops to almost zero at ≈9 L, it indicates that virtually all (3 × 3) domains are converted to h-BN.

The temperature region where the BN_I_ structure evolves is in excellent agreement with the borazine decomposition reported by Paffett *et al.*,^40^ which helps to confirm the dehydrogenation process. Further, as mentioned earlier, bulk h-BN is known to form by a sequence of dehydrogenation processes, in which borazine polymerises to polyborazylene, which is then cross-linked in one or more steps.^10,11^ Our results suggest that a similar process happens at the ruthenium surface, but that in the 2D case, there is one clear intermediate step, *i.e.* the BN_I_ structure, before the formation of h-BN. There are several possible real space structures, which are in line with the observed periodicity and composition.^23^ We concentrate on providing experimental proof that such intermediate/precursor structures with long-range order exist, while the exact chemical composition of these structures is better probed with X-ray photoemission spectroscopy (XPS) studies,^41^ beyond the mentioned structural analysis. Moreover, the precursor gas imposes a B/N ratio of 1 : 1 which we do not expect to change and we have therefore focussed on supporting our structural characterisation through detailed DFT modelling of appropriate candidate structures.

### DFT structural modelling

We understand the BN_I_ structure to consist of partially dehydrogenated, polymerised borazine analogous to the synthesis of bulk h-BN. Thus it is apparent that the 2D growth occurs in a step-by-step process and that not all hydrogen atoms are expected to be removed at the same time, due to the different bonding strengths to N and B atoms, as well as the bond formation between the N and the Ru atoms.^41,42,44^ Based on this attribution, we have made a vdW-corrected DFT investigation into the energetics of the BN_I_ structure, from adsorption of the precursor gas to the complete h-BN overlayer.

We start by considering a single borazine molecule in a (3 × 3) supercell (see Computational methods in the ESI†), and move on to partially dehydrogenated borazine polymers. From the adsorption energies of isolated borazine molecules we observe that the bonding becomes much stronger with dehydrogenation, but the calculations cannot provide a definitive answer in terms of the dehydrogenation sequence (dehydrogenation of the B atoms is slightly more favourable than of the N atoms by ≈ 15 meV). However, as shown later for two borazine molecules per supercell, the N atoms can dehydrogenate more easily than the B atoms and the candidate structures for our observations can be clearly distinguished in terms of the adsorption energies.

**Fig. 4:**
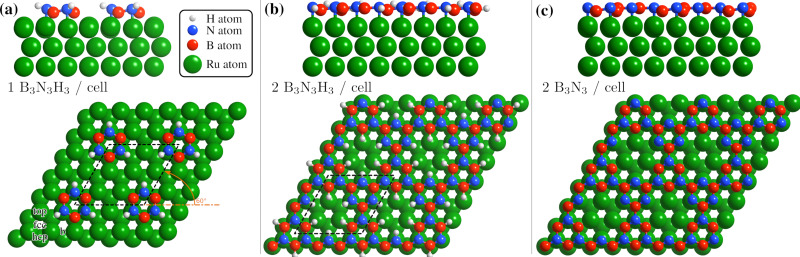
Side and top view of the energetically most favourable configurations on the Ru(0001) surface, from one to two borazine molecules per supercell. Dehydrogenation occurs in stages^40–42^ with bonding to the substrate facilitated *via* the N atoms^6,24^ and we thus expect that dehydrogenation starts at the site of the B atoms for two or more molecules per supercell, as also confirmed by the DFT calculations (see text). (a) For one partially dehydrogenated borazine molecule (B_3_N_3_H_3_) adsorption occurs on the fcc site, forming a (3 × 3) structure. (b) Shows the structure for two partially dehydrogenated B_3_N_3_H_3_ molecules per supercell, where bound B–N rings form a nanostructured network and hydrogen atoms remain adsorbed on the Ru lattice inside the nanopores. In (c) the optimised structure for two fully dehydrogenated borazine molecules is shown, leading to the same nanopore structure with a (3 × 3) periodicity.

In Table 1 we compare the binding energies from vdW-corrected DFT for an intact (B_3_N_3_H_6_) and a partially dehydrogenated (B_3_N_3_H_3_) borazine molecule with one molecule per (3 × 3) supercell, confirming a much stronger bonding of B_3_N_3_H_3_. We consider various initial adsorption sites (Fig. 4(a)) with respect to the *C*_3_ rotational axis through the centre of the molecule and a rotation of 60°. Adsorption occurs in a flat face-to-face configuration, while bonding of the same adsorbates with a rotation of 0° is slightly weaker – the results are shown in the ESI† (see Supplementary DFT calculations).

**Table 1:** DFT calculations for the adsorption structures of the borazine precursor on Ru(0001), based on one molecule per (3 × 3) supercell. The results are shown for an intact (B_3_N_3_H_6_) and partially dehydrogenated (B_3_N_3_H_3_) adsorbate, considering various initial adsorption sites and a rotation of 60° (see Fig. 4(a)). The adsorption energies *E*_ads_ are given for the final optimised adsorption sites and Δ*E* is the difference with respect to the minimum energy configuration of the system with the same dehydrogenation state

B_3_N_3_H_6_			B_3_N_3_H_3_		
Site	*E*_ads_ (eV)	Δ*E* (eV)	Site	*E*_ads_ (eV)	Δ*E* (eV)
fcc	−4.08	0.00	fcc	−8.95	0.00
top	−1.21	2.86	top	−6.60	2.35
b → fcc	−4.08	0.00	b → fcc	−8.95	0.00
hcp	−1.31	2.77	hcp	−5.88	3.07

In Table 1 the energy differences Δ*E* are given with respect to the minimum energy of the same dehydrogenation state in addition to the respective adsorption energies *E*_ads_. For both stoichiometric configurations the most favourable position is the fcc site and if the borazine molecule is initially placed on a bridge site it undergoes a transition to this position. The fcc configuration for partially dehydrogenated borazine yields an adsorption energy of *E*_ads_ = −8.95 eV and is shown in Fig. 4(a) with the (3 × 3) supercell highlighted by the black dashed rhombus. The results for the intact borazine molecule (B_3_N_3_H_6_) are very similar with respect to the adsorption site, however, we obtain significantly weaker bonding strengths compared to the dehydrogenated molecule.

Based on bulk h-BN studies we conclude that it is more likely that polymerised networks are formed.^10,11^ Starting from the minimum energy configuration of a single borazine molecule on the fcc site we continue by adding a second borazine molecule in the supercell. By considering various initial rotations of the additional molecule the energetically most favourable configurations were then identified. In contrast to the case of an isolated borazine molecule, the dehydrogenation sequence becomes clearly discernible in terms of the adsorption energies, with two borazine molecules. The N atoms in the ring adsorb on top of the Ru atoms and the borazine molecules lose all hydrogen atoms associated with the N atoms upon bond formation, in line with experimental results of the completed h-BN overlayer where inter-layer bonding is facilitated *via* the N atoms.^6,24^ Such a scenario is, however, different to bulk h-BN growth where ruthenium is not present and thus interaction with the substrate may give rise to an even faster loss of hydrogen compared to bulk studies.

The calculations for two intact borazine molecules per supercell (2B_3_N_3_H_6_, not shown) yield weak binding, since the H atoms start to overlap resulting in a tilt of the complete molecules with respect to the surface. Moreover, due to desorption of hydrogen atoms from the borazine at low temperatures it is unlikely that intact borazine will remain and so intact molecules will not be considered further.^40^

Therefore, we concentrate on partially and fully dehydrogenated borazine molecules. Fig. 4(b) and (c) shows the final optimised structure for 2B_3_N_3_H_3_ per supercell, illustrating that individual borazine molecules form bonds to each other. The bound B–N rings build up a nanostructured network with nanopores, *i.e.* where in between the B–N rings vacancies/pores of the Ru substrate are left behind. The high binding energy of the structure in Fig. 4(b) with −6.28 eV compared to −6.74 eV for the complete h-BN/Ru, may therefore explain the stability of the BN_I_ structure at temperatures ≈ 750 K as observed in the experiments.

The structure in Fig. 4(b) acts as an intermediate prior to complete dehydrogenation which is expected at elevated temperatures. The calculations show that first the hydrogen atoms detach from the nitrogen and bind to the Ru substrate on the hcp sites, inside the nanopores. The excess hydrogen adatoms inside the nanopores are likely to desorb relatively quickly at the temperature of the experiment.^63^ Therefore, Fig. 4(c) shows the optimised structure, starting with two fully dehydrogenated borazine molecules per supercell, leading again to the formation of nanopores. Such an open structure could easily act as a precursor to the complete h-BN overlayer, since each pore only has to be “filled” with an additional dehydrogenated borazine molecule. Finally, the addition of further borazine molecules in the calculations, *i.e.* three per supercell, essentially leads to the formation of h-BN which gives rise to the strongest binding energy in the calculations. The route from the precursor BN_I_ structure to the final h-BN overlayer, with several intermediate steps, is illustrated in Fig. S3 of the ESI.†

In addition to providing us with real-space structures of the observed BN_I_ precursor, there are several points which we note from the vdW DFT calculations: dehydrogenation of borazine always gives rise to a stronger bonding to the substrate and the results show that the thermodynamically most stable configuration for three adsorbed borazine molecules is h-BN (Fig. S2(b), ESI†). We also see from the side views in Fig. 4 that there occurs always some buckling (0.21–0.35 Å) and the adlayer is never perfectly flat. The results show that by carefully controlling the substrate temperature and thus the amount of excess hydrogen in future experiments, several BN nano-structures could be synthesized as shown for two cases in Fig. 4(b) and (c). Moreover, careful changes of the starting conditions in the DFT calculations may even yield a “local” minimum energy configuration as in Fig. S2(c) (ESI†). Thus the system may be an ideal playground for the growth of different nano-structures and further metastable networks beside the ones reported in this work.

### Additional structures accompanying the h-BN growth

So far, we have described the formation of a BN_I_ structure at *T* ≥ 750 K, which is converted to h-BN at *T* ≥ 880 K. However, upon complete conversion of the BN_I_ structure to h-BN, exposing the surface to excess borazine results in the emergence of an additional structure with a (3 × 4) periodicity, which we label BN_II_. The green line in Fig. 2(b) illustrates the corresponding diffraction pattern with the h-BN Moiré diffraction peak being still present next to the first order Ru peak. As shown in a two-dimensional diffraction scan in Fig. S5 (ESI†), the (3 × 4) peaks are not a subset of the h-BN Moiré pattern. In addition, a smaller peak to the left of the first order Ru peak becomes visible which can be attributed to a substrate reconstruction peak^29^ due to the h-BN growth.

**Fig. 5:**
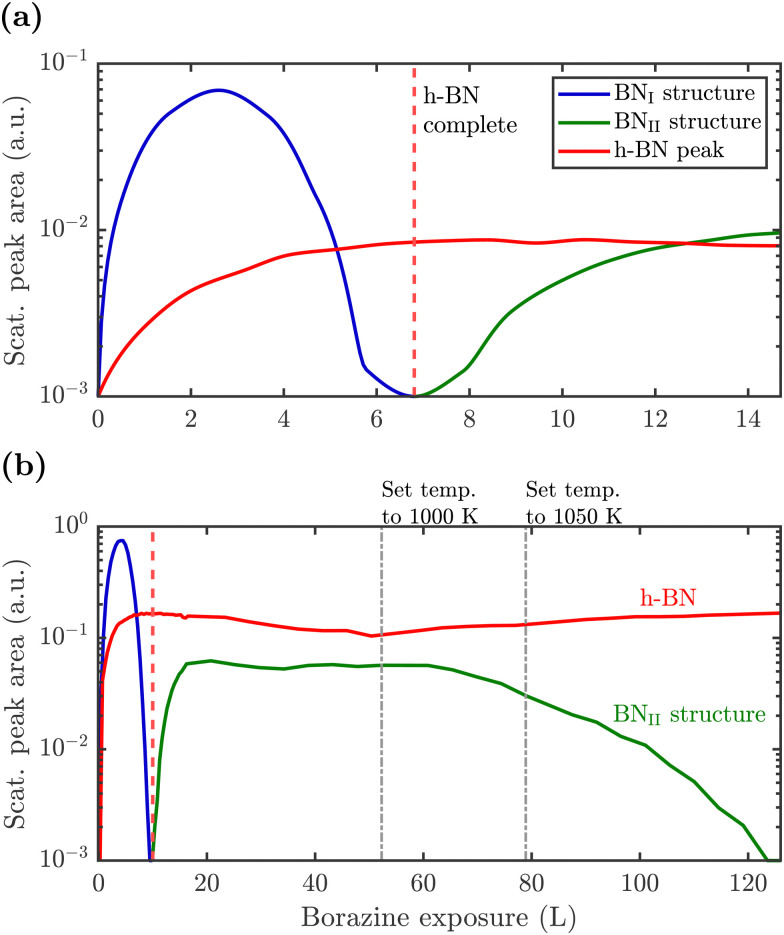
Peak areas of the characteristic diffraction peaks representing the different structures *versus* borazine exposure. After the BN_I_ structure has disappeared the h-BN peak saturates giving rise to a conversion and further exposure leads to the rise of the BN_II_ structure. Due to a higher substrate temperature of 915 K in (a), the BN_I_ structure disappears already after an exposure of ≈7 L compared to 10 L at 880 K in (b), thus indicating a kinetically driven conversion. Dosing in (b) is then further continued with subsequent changes of the surface temperature as stated above the diagram. After long enough exposure the BN_II_ structure disappears leaving a strong h-BN intensity behind.

To monitor the growth of the BN_II_ structure we use a smaller borazine overpressure while holding the sample temperature at 915 K. Fig. 5(a) shows the evolution of the BN_II_ and the BN_I_ structure as blue and green curves, respectively. Here, the red line is again the integrated peak intensity of the h-BN diffraction peak. Immediately after exposing the surface to borazine, the BN_I_ structure increases together with the h-BN peak. Further exposure leads to a decay of the BN_I_ structure, while the h-BN feature still rises, indicating the growth of h-BN islands. At 7 L the h-BN diffraction peak saturates, while at the same time the BN_I_ structure disappears. At this stage the h-BN overlayer is complete and after further dosing of borazine, the BN_II_ structure starts to emerge. As discussed later this may be interpreted as a second layer being formed on top of h-BN.

The measurement was repeated at an even lower dosing pressure, while holding the sample at the lower temperature of 880 K. In Fig. 5(b) the same behaviour is reproduced, yielding a h-BN layer with two additional structures, except that the emergence is delayed to longer/higher exposures, thus indicating a kinetically driven conversion.

With continuing borazine exposure to 20 L in Fig. 5(b), the BN_II_ structure reaches its maximum with no further changes in the scattered intensity. Together with the rise of the BN_II_ structure the h-BN peak intensity slowly starts to decay, likely due to diffuse scattering from additional adsorbates at the surface or from domain walls of the BN_II_ structure. Increasing the surface temperature to 1000 K gives rise to a decay of the BN_II_ structure while the h-BN peak intensity starts to recover to its original value. Further temperature increase accelerates this process giving rise to a faster transition/conversion until the intermediate peaks disappear, leaving behind only the h-BN layer. Such a behaviour illustrates that ultimately h-BN is the most stable structure. Even though the borazine overpressure was still present, no additional peaks formed and the h-BN overlayer is the only remaining structure at the surface.

From Fig. 5(b) it becomes evident, that in contrast to the BN_I_ structure, the BN_II_ structure is much more stable at higher temperatures since the (3 × 4) diffraction peaks are observed up to 1000 K. Further increase of the temperature to ≈1200 K gives rise to the surface migration of bulk-dissolved carbon, leading to the formation of graphene (see Supplementary diffraction scans in the ESI†) and thus eventually destroys the h-BN overlayer. The latter may open up the possibility to study the growth of h-BN/graphene heterostructures.^64–67^

From our experiments it is likely that the BN_II_ structure is a second chemisorbed layer on top of already grown h-BN. Earlier works on an Ir(111) substrate showed the evolution of additional compact reconstructed regions with a (6 × 2) superstructure, which were attributed to reconstructed boron areas.^68^ On the other hand, CVD growth on polycrystalline Cu provided evidence for boron dissolution into the bulk together with multilayer h-BN formation *via* intercalation.^69^ However, both systems and studies are significantly different from our approach. *E.g.*, the different behaviour in the first study could be due to changes of both the lattice constant and the h-BN-substrate bonding between Ru and Ir. Moreover, in light of the recent observation of h-BN multilayer growth,^3^ a second chemisorbed layer is much more plausible.

The BN_II_ structure, as a second chemisorbed layer, consists of partly or completely dehydrogenated borazine molecules with a desorption temperature slightly above our performed measurements, since we see an adsorption/desorption equilibrium at temperatures ⪅1000 K with an ultimate desorption at temperatures above this value. We can rule out the existence of multilayer growth due to the absence of intensity oscillations in our experiments^32^ or observations of any other periodicity, that would be indicative of multilayer h-BN growth. On the other hand, considering different growth conditions at higher pressures or *via* boron dissolution in the substrate,^3^ the BN_II_ structure may well act as a precursor to multilayer synthesis, in particular if the original h-BN layer is of poor quality thus providing a high density of growth nuclei and further facilitating multilayer growth.^3,69–72^

In a set of additional DFT calculations summarised in Table S2 of the ESI,† we considered also the possibility of borazine adsorption on top of h-BN/Ru as well as the formation of bi-layer h-BN.^69–72^ As mentioned above, we can rule out the latter at our experimental conditions. Thus it is more likely that the BN_II_ structure consists of adsorbed molecules or polymerised borazine structures, with a weaker bonding compared to the first h-BN layer^3^ and therefore more likely to desorb, in line with the multi-stage process of h-BN bulk formation. Further unlikely scenarios are discussed in Supplementary discussion in the ESI.†

### h-BN growth diagram on Ru(0001)

**Fig. 6:**
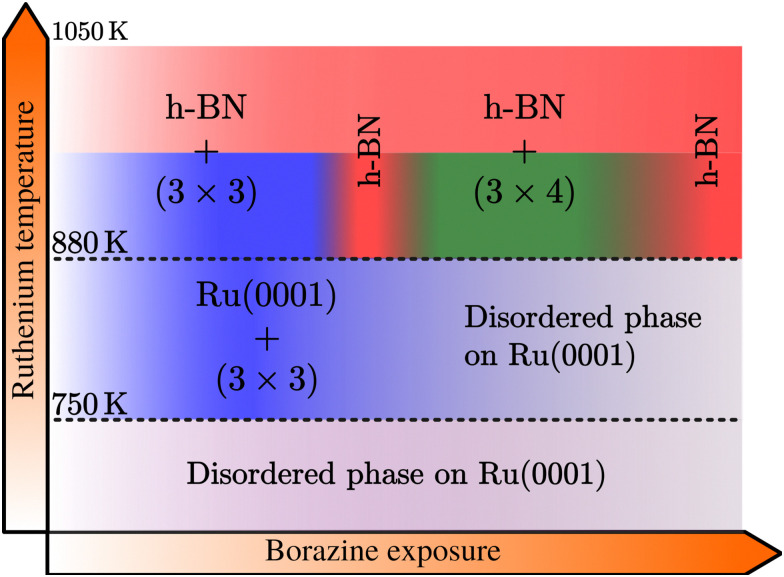
Schematic growth diagram of the different BN structures observed on a Ru surface within various temperature ranges and with increasing borazine exposure. At temperatures below 750 K no periodic structure is formed, while above, a (3 × 3) pattern is observed, disappearing again at higher exposures. Above 880 K the (3 × 3) structure precedes the growth of h-BN, with the temperature being necessary for the formation of h-BN. With increasing borazine exposure, the latter is followed by a structure with (3 × 4) periodicity with respect to the Ru lattice, leading again to h-BN after long enough exposure. (See text for a precise explanation of the structures.)

The combination of measurements and DFT calculations allows us to conclude that the whole system passes through various structural phases:Ru + BZ → BN_I_ + h-BN → h-BN → BN_II_ + h-BN → h-BN,with the outcome depending strongly on substrate temperature, borazine exposure and the point where one stops. In particular, the surface temperature strongly influences the kinetics and thus the duration and appearance of the additional superstructures. Combining the experimental results we derive a growth diagram as shown in Fig. 6, which describes the phenomenology of various structures arising during the CVD growth of h-BN on Ru(0001).

Below 750 K no periodic overlayer structure on the Ru(0001) surface is found. Between 750 and 880 K the BN_I_ structure forms on the surface which upon further borazine exposure vanishes and leaves a disordered phase behind. The minimum temperature to form a h-BN overlayer on the surface was determined to be 880 K. Above this temperature we observe additional structures, starting with a (3 × 3) structure (BN_I_) followed by a (3 × 4) periodic diffraction pattern (BN_II_). These structures always appear in addition to the h-BN layer and ultimately vanish, leaving a complete h-BN overlayer behind (see Fig. 2(a) for a diffraction scan of a complete h-BN overlayer without any additional structures). We have thus identified two kinetic barriers which need to be overcome in order to form ordered structures on the Ru substrate: a temperature of 750 K is necessary for the precursor structure to result, while at 880 K the h-BN formation sets in. As mentioned above, the BN_I_ precursor structure is always present, however, with increasing temperature its transformation into h-BN becomes faster.

As further illustrated in h-BN periodicity in the ESI,† the h-BN periodicity and superstructure are strongly dependent on the experimental parameters, in particular the growth temperature. The exact h-BN periodicity and the Moiré pattern upon h-BN formation on Ru(0001),^24,26^ due to the small lattice mismatch between *a*_h-BN_ and *a*_Ru(0001)_, is determined by the growth temperature due to a so-called “lock-in” effect at that respective growth temperature.^73^ Together with the above reported additional structures, it confirms the complexity of the whole system and its dependence on minute changes of the growth parameters.

## Discussion and summary

In summary, we investigated the growth of h-BN on a Ru(0001) substrate using helium atom scattering. Employing various growth conditions, characteristic periodic structures are measured during borazine exposure in addition to the h-BN diffraction peak as outlined in the diagram of Fig. 6. Between 750 and 880 K a structure with (3 × 3) periodicity, that precedes the growth of h-BN, is observed with the minimum temperature necessary to form a h-BN overlayer being 880 K. Above this temperature, in addition to the emerging h-BN layer, we observe additional structures with a (3 × 3) superstructure followed by a (3 × 4) diffraction pattern, eventually disappearing and leaving a complete h-BN overlayer behind.

It is clearly evident from our observations that a precursor structure precedes the growth of h-BN at lower temperatures and an additional structure co-exists with h-BN at higher temperatures. Both are strongly dependent on the growth conditions, but always transform into a fully h-BN covered substrate at sufficiently high temperatures, thus confirming that the latter is the thermodynamically most stable structure. Our study of the structural evolution during the arrangement of h-BN from the precursor gas illustrates steps in the formation process itself and we hope to encourage future studies linking our structural information with chemical characterisation.

We believe that these intermediate metastable structures may be present in many more systems where 2D materials are grown based on CVD, at least at lower temperatures and for higher amounts of excess hydrogen compared to the “ideal” growth conditions (see Outlook for other 2D materials in the ESI†). In the case studied here, they ultimately always transform into the complete 2D layer – and thus usually higher temperatures are reported as the “ideal” growth conditions for h-BN in the literature.

These intermediate structures seem to have been largely overlooked so far. Possibly, because they are difficult to detect owing to experimental complications since the structural advent of 2D materials is often not investigated during the growth itself, or is only accessible *ex situ*. More importantly, with increasing growth temperature the transformation to h-BN may occur so fast that they are easily missed.^27^

The strong dependence regarding the emergence of these structures on temperature and exposure suggests that further uncovered “routes” and polymerisation steps are viable and the system may present an ideal playground to end up with different nano-structures. It further suggests that a careful tuning of the growth conditions *via* temperature and excess hydrogen from the precursor may provide new broadly applicable strategies for controlling the growth of specific nanostructures. Additional possibilities involve changing the substrate or the precursor gas, and hence tuning the thermochemistry of the surface–adsorbate complex which may further alter the subsequent reaction pathway. *E.g.* by changing the substrate, the metal–N bond strength may be tuned since one expects the bonding strength to increase as one moves from right to left in the transition metal series. We hope that the wide ranging implications for a controlled growth of 2D materials and nanostructures will stimulate a broad range of new research, understanding and application.

## Author contributions

A. R. and N. A. carried the diffraction measurements out, with M. S. and A. P. performing the *ab initio* (DFT) calculations. A. T. and A. R. worked on the structural analysis, while A. T., A. R., M. S. and N. A. developed the physical interpretation of the data. A. P. J. and W. E. contributed to the conception of the project and all authors discussed the results and contributed to writing the manuscript.

## Data availability

The datasets generated and analysed during the current study are available from the TU Graz repository, with the identifier DOI: 10.3217/1hwgj-rgg36.

## Conflicts of interest

The authors declare no competing financial interest.

## Supplementary Material

NH-007-D2NH00353H-s001
