# Supplementary material for: Evolution of ordered nanoporous phases during h-BN growth: controlling the route from gas-phase precursor to 2D material by in situ monitoring
Source: Nanoscale Horiz. 2022 Sep 21;7(11):1388–96. doi: 10.1039/d2nh00353h (PMC9590587; doi:10.1039/d2nh00353h)
Supplement: NH-007-D2NH00353H-s001 [file NH-007-D2NH00353H-s001.pdf]

Electronic Supplementary Material (ESI) for Nanoscale Horizons.
This journal is © The Royal Society of Chemistry 2022

Supplementary Information for

Evolution of ordered nanoporous phases during h-BN growth: Controlling the route from gas-phase precursor to 2D material by *in-situ* monitoring.

1 Experimental Section

All measurements were performed with the ^{3}He spin echo apparatus at the Cambridge Atom Scattering Centre. A schematic of the scattering chamber in the experimental setup is shown in Supplementary Figure 1. The helium beam is produced by supersonic expansion of ^{3}He gas through a nozzle and enters the scattering chamber through a series of differential pumping stages. The incident helium beam is scattered off the sample, which is, together with a sample holder, mounted on a 6-axis manipulator.[1] Atoms travelling in a particular outgoing direction pass along the second arm of the instrument, at 44.4° total scattering angle, and are then ionised and counted in a high sensitivity mass-spectrometer detector. The incidence angle, ϑ_i, with respect to the surface normal, can be varied to control the momentum transfer on scattering. The

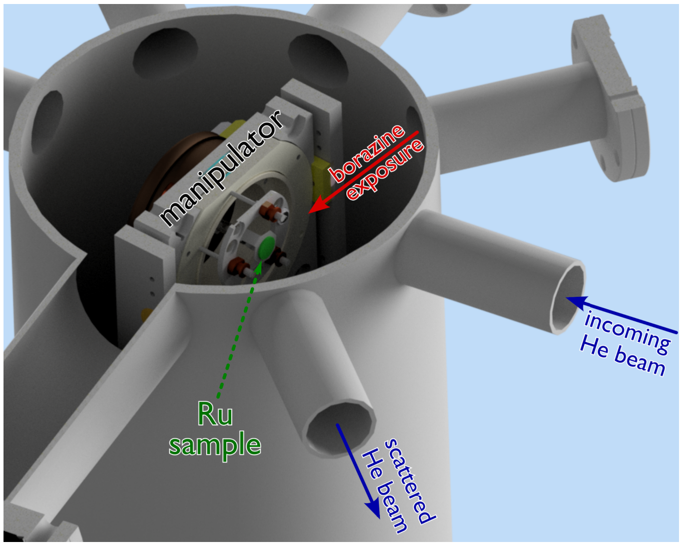

Supplementary Figure 1 Schematic of the experimental setup in the helium scattering chamber. The incoming He beam is scattered off the ruthenium (Ru) sample in a fixed source-detector configuration with an angle of 44.4°. The sample is mounted onto a 5-axis manipulator and can be exposed to borazine via dosing thorough a leak valve.

Ru sample can be heated radiatively and by electron-bombardment from backside of the crystal and cooled via a thermal connection to a liquid nitrogen reservoir. The entire beamline is held at high vacuum to avoid any attenuation of the helium beam, and the sample and detector chamber require ultra high vacuum levels to maintain cleanness of the sample and a low ^{3}He background. The dosing was performed by backfilling the scattering chamber with borazine vapour, with borazine as provided by Katchem. To monitor the dosing rates, the chamber pressure was monitored, with typical overpressures between 1×10^{-9} and 5×10^{-8} mbar. At stages where borazine was not used for dosing the container was held at temperatures below 0 °C.

The Ru(0001) surface was cleaned by Ar-sputtering and annealing to 1300 K with subsequent O_2 treatment to not less than 20 L at 700 K. The adsorbed O_2 was removed by repeated flashing cycles to 1200 K. The cleanliness of the sample was determined by helium reflectivity measurements and diffraction scans to show no features of adsorbed species. After reaching reflectivities of $\approx 23\%$ the sample was ready for the various dosing conditions. h-BN overlayers were removed by oxygen treatment at a sample temperature of 900 K, followed by the cleaning explained above. Borazine was supplied to the sample by backfilling the chamber through a leak-valve with typical overpressures between 1×10^{-9} and 5×10^{-8} mbar.

2 Computational Methods

For the DFT calculations we employed CASTEP,[2] a plane wave periodic boundary condition code. The plane wave basis set was truncated at an electron energy cut-off of 400 eV and we employ Vanderbilt ultrasoft pseudopotentials.[3] The Brillouin zone was sampled with a $(4 \times 4 \times 1)$ Monkhorst-Pack k-point mesh.[4] The Perdew Burke Ernzerhof exchange correlation functional[5] was applied in combination with the Tkatchenko and Scheffler dispersion correction method.[6] The Ru(0001) surface was modelled by a 5-layer slab in a (3×3) supercell, and an additional 15 Å vacuum layer for separating the periodically repeated supercells in the z-direction. Positions of the atoms in the adsorbate and in the top three layers of the Ru substrate were left fully unconstrained. For the structural optimisations, the force tolerance was set to 0.05 eV/Å.

The adsorption energies E_{ads} are defined to be:

$$E_{ads} = E_{tot}(x + ny) - E_{tot}(x) - nE_{tot}(y)$$

where $E_{tot}(x+ny)$ is the total energy of the system, $E_{tot}(x)$ is the energy of the substrate, $E_{tot}(y)$ is the energy of the adsorbate and n is the number of adsorbed molecules. The more negative E_{ads}, the more thermodynamically favourable it is for the species to adsorb.

In order to compare the intermediate structures with a different number of atoms we calculate the binding energy E_{bin} relative to Ru(0001) + 3 borazine molecules (3 borazine molecules are needed to form h-BN on a (3×3) cell) by appropriately adding or subtracting the energy of H_2 and borazine in the gas phase, to preserve stoichiometry:

$$E_{bin} = E_{tot} + \frac{n_H}{2} E_{tot}(H_2) + n_{BZ} E_{tot}(BZ) - E_{tot}(Ru) - E_{tot}(3BZ)$$

where E_{tot} is the total energy of the system, $E_{tot}(H_2)$ and $E_{tot}(BZ)$

are the energies of H_2 and borazine which remain in the gas phase, respectively and $E_{tot}(Ru)$ and $E_{tot}(3BZ)$ are the total energies of pristine Ru(0001) and 3 borazine molecules in the gas phase. The more negative E_{bin}, the stronger the binding and it becomes thermodynamically more favourable for the species to form.

3 Supplementary DFT calculations

The energetically most favourable adsorption site for a single intact borazine molecule per (3×3) supercell according to DFT calculations is shown in Supplementary Figure 2(a). The adsorption sites (top, hcp, fcc, b) are given relative to the centre of the borazine molecule. In addition to the calculations for one borazine molecule

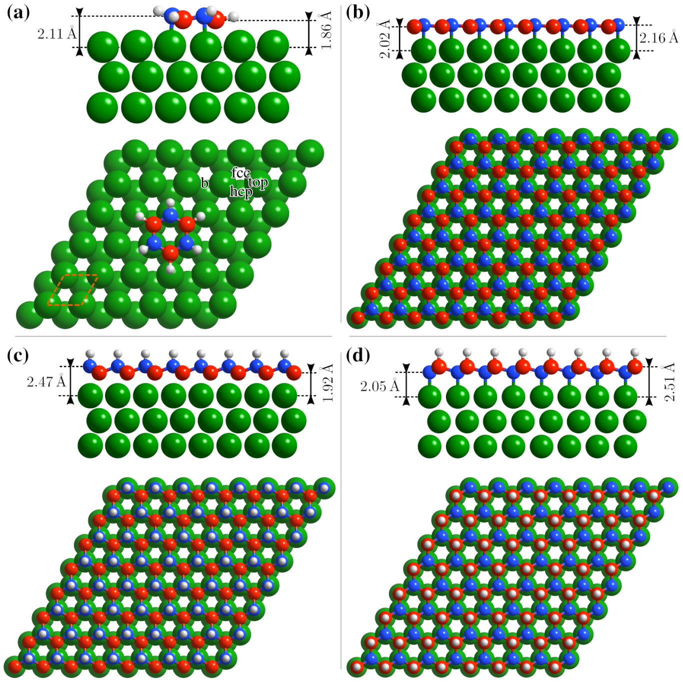

Supplementary Figure 2 (a) depicts the outcome of vdW-corrected DFT calculations for one borazine molecule ($B_3N_3H_6$) on Ru(0001), with the most favourable adsorption site being the fcc position. All possible adsorption sites are given with their according labels. (b) shows the top and side-view of h-BN on Ru(0001) with the corresponding interatomic distances. In (c) and (d) the optimised geometry for three partially dehydrogenated borazine molecules is illustrated, which essentially forms a hydrogenated version of h-BN/Ru(0001). Here (c) represents a "local" minimum as also seen from Supplementary Table 2. The hydrogen atoms stick out of the surface, yielding a high corrugation and hence the buckling will be different once the structure is dehydrogenated, such as for a complete h-BN layer with its corrugation reflecting the Moiré pattern.

given in the main text we show the results for the intact and partly dehydrogenated molecule with an initial rotation of 0° in Supplementary Table 1. When comparing the results we now see that in this case the hcp site is energetically most favourable with an adsorption energy of $E_{ads} = -8.85$ eV. If the borazine molecule is initially placed on a bridge site it undergoes a transition to the hcp position. Still, the results for the 60° rotation are energetically more favourable by ≈ 0.1 eV.

Supplementary Figure 2(c,d) shows that the outcome for calculations considering three partially dehydrogenated borazine molecules on Ru(0001), results in a structure similar to h-BN, except for the fact that the H atoms remain attached to the boron/nitrogen atoms. For comparison, Supplementary Figure 2(b) depicts the optimised structure for h-BN/Ru(0001).

Supplementary Table 2 illustrates that hydrogenation of the h-BN overlayer becomes thermodynamically unfavourable due to the correction with respect to molecular hydrogen in the gas phase and the high binding energy of the latter. The result is in line with hydrogenation experiments of metal supported h-BN, where atomic hydrogen exposure is required in order to facilitate the hydrogenation.[7] Interestingly, in contrast to h-BN/Ni(111),[7] H adsorption on top of the N-site is slightly more favourable than on top of the boron site for h-BN/Ru(0001) as can be seen from the adsorption energy per hydrogen atom.

Supplementary Table 1 DFT calculations for the adsorption structures of the borazine precursor on Ru(0001), based on a (3×3) supercell with one molecule per cell. The results are shown for an intact ($B_3N_3H_6$) and partially dehydrogenated ($B_3N_3H_3$) adsorbate, considering various initial adsorption sites and a rotation of 0°. The adsorption energies E_{ads} are given for the final optimised adsorption site and ΔE is the difference with respect to the minimum energy configuration of the system with the same dehydrogenation state.

$B_3N_3H_6$			$B_3N_3H_3$		
Site	E_{ads} (eV)	ΔE (eV)	Site	E_{ads} (eV)	ΔE (eV)
fcc	-1.28	2.80	fcc	-5.96	2.99
top	-1.21	2.87	top	-6.60	2.35
b→hcp	-3.99	0.08	b→hcp	-8.85	0.11
hcp	-3.99	0.08	hcp	-8.85	0.11

From the side view in Supplementary Figure 2(c,d) it becomes evident that the closest atom to the Ru substrate and the bond length change, depending whether nitrogen or boron remain hydrogenated. In Supplementary Figure 2(d) the hydrogen atoms appear to "pull" the boron away from the surface by 0.5 Å and the sp^2 hybridised bonds to nitrogen gain more sp^3 character. Therefore the boron atom moves away from the surface to optimise these bonds, forming a tetrahedral (bond angle 106°). Likewise the nitrogen binds to the Ru orbitals, thus moving closer to the surface. If hydrogen desorbs from this structure pure h-BN is formed, as seen in Supplementary Figure 2(b). The boron-nitrogen bonds become stronger and therefore boron moves 0.5 Å towards the Ru, to be in the same plane as the nitrogen. In addition the nitrogen orbitals are populated from the boron and the nitrogen-Ru interaction is weakened, resulting in a movement of the nitrogen atoms 0.11 Å away from the Ru surface. For pure h-BN on Ru, the boron atoms are positioned only slightly lower than the nitrogen atoms (0.14 Å). This may reflect the gain in stability from Ru-B bonding when boron is moved slightly into the hole site, compared to maintaining perfect sp^2 hybridised bonds.

As mentioned in the main text, we considered also borazine adsorption on top of h-BN/Ru as well as the formation of bi-layer h-BN. The physisorption energies are shown in the lower part of Supplementary Table 2, illustrating that both are thermodynamically favourable with a stronger physisorption energy for a

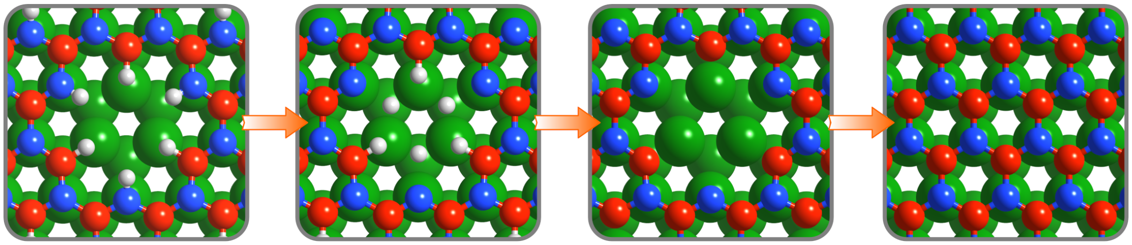

Supplementary Figure 3 Schematic of the possible route from the partially dehydrogenated precursor structure to h-BN via several intermediate structures based on vdW-corrected DFT calculations. As noted in the text, the precursor structure is already quite close to the binding energy for the complete h-BN layer. In contrast to the DFT calculations, where entropy contributions were not considered, additional dehdrogenation and bond breaking may occur due to the high experimental temperatures.

Supplementary Table 2 DFT calculations for different configurations/structures with their respective energies.

Configuration	Adsorption energy per H atom (eV)	Adsorption site
hydrogenated h-BN/Ru	0.84	H on top of B
hydrogenated h-BN/Ru	0.81	H on top of N

Configuration	Physisorption energy (eV)	Stacking
h-BN on h-BN/Ru	-2.09	AB
Borazine on h-BN/Ru	-0.73	AB

second h-BN layer on top of h-BN/Ru. On the other hand, the corresponding binding energy for a single complete h-BN layer is -6.74 eV upon formation from 3 borazine molecules per supercell on Ru(0001). In the following we consider a possible route to the complete h-BN layer starting from the precursor structure as described in the main paper,

Supplementary Figure 3 shows the route through various steps based on DFT calculations. The first (precursor) structure is strongly bound with a binding energy E_{bind} (see Computational methods) of -6.28 eV in relation to the bare Ru surface and the molecules in the gas phase. The next step towards h-BN formation, involves dehydrogenation. The calculations show that if the three hydrogen atoms are detached only from the boron atoms they eventually reattach to the same boron sites. Therefore, initially the hydrogen atoms are detached from the nitrogen atoms which adsorb on the Ru substrate within the nanopores, yielding a binding energy of -3.27 eV. At sufficient surface temperature eventually all hydrogen atoms will desorb from the surface yielding the third structure with a less favourable energy of 1.26 eV. If now the nanopore is filled with one additional borazine atom, h-BN is formed yielding the lowest binding energy (-6.74 eV). Therefore we conclude that the first structure is nearly as stable as h-BN and that on the route to h-BN several energy barriers have to be overcome. It should be mentioned however, that the calculations were performed at 0 K and that no entropy contributions were considered.

4 Supplementary diffraction scans

h-BN, sometimes also called “white graphene”, typically forms a Moiré pattern on the surfaces of reactive transition metals such as Rh(111) or Ru(0001), as mentioned in the main text. The two-dimensional h-BN layer on such surfaces exhibits periodic nanometric structures, often called “nanomesh”, with areas which are elevated from the surface, and areas closer to the surface. In Supplementary Figure 4 the characteristic diffraction pattern of the clean Ru sample (green) is compared to two overlayers on the same substrate.

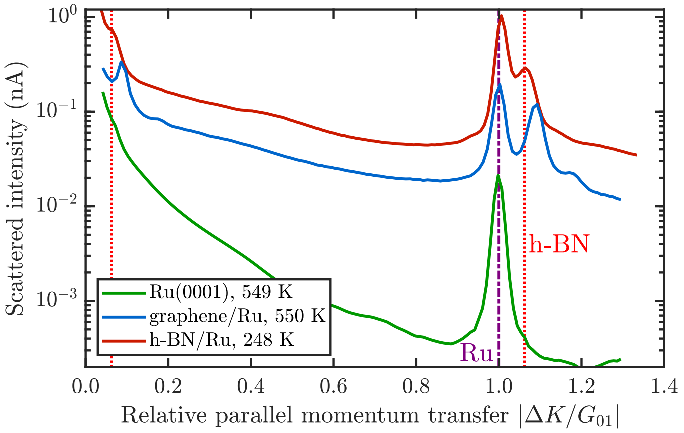

Supplementary Figure 4 Angular diffraction patterns using helium scattering. Comparison of the diffraction scans of the clean Ru(0001) surface (green), graphene on Ru (blue) and h-BN on Ru. The purple dash-dotted line indicates the first order diffraction position and the red dotted lines the h-BN reconstruction peaks.

The scans of the single layer graphene and h-BN covered Ru show additional peaks close to the specular and first order Ru diffraction peaks. The blue curve depicts the scattering result for a graphene monolayer on Ru which has been studied extensively in earlier works.[8,9] The graphene layer was grown by heating the Ru crystal to 1250 K for several minutes. Leaving the crystal at such high temperatures brings the carbon out of the bulk which then forms the honeycomb single layer graphene sheet. Graphene forms a (12-on-11) superstructure in which a (12×12) supercell of

graphene coincides with a (11×11) supercell of ruthenium, giving rise to additional diffraction peaks at $|\Delta K/G_{01}| = 1/11 \equiv 0.09$ and $|\Delta K/G_{01}| = 12/11 \equiv 1.09$.

The diffraction pattern for h-BN on a Ru substrate is depicted in red in Supplementary Figure 4. Earlier works indicate that h-BN forms a (13-on-12) superstructure which can be identified by position of the diffraction peaks.[10] Indeed the feature originating from the h-BN nanomesh to the right of the Ru diffraction peak shifts to smaller values of $|\Delta K/G_{01}|$ with respect to graphene, giving rise to a bigger supercell.

In Supplementary Figure 4 the scans for pure Ru and graphene were performed at a sample temperature of $T = 550\,\text{K}$ while the scan of h-BN was taken at 248 K. Due to thermal expansion it gives rise to a deviation of the position of the first order substrate (Ru) peak for the h-BN scan compared to the other two measurements as indicated by the purple line. In all scans the specular peak (at $|\Delta K/G_{01}| = 0$) was cut off due the high intensity and the first order diffraction peak of the Ru surface corresponds to $|\Delta K/G_{01}| = 1$.

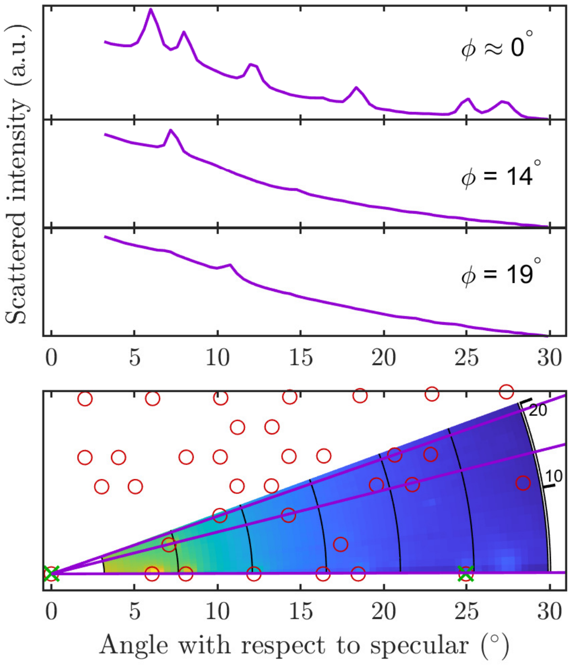

Supplementary Figure 5 2-dimensional scan of the (3×4) structure of the adsorbed borazine molecules on the Ru surface. The polar plot consists of 22 individual logarithmic diffraction scans at various azimuthal orientations φ and a surface temperature of 300 K. The red circles indicate the calculated scattering positions for a (3×4) superstructure while the green crosses mark the Ru diffraction positions. Three exemplary scans at the top are drawn to elucidate the diffraction peak positions in dependence of φ.

In addition Supplementary Figure 4 clearly shows that the background intensity between the Ru diffraction peaks is much lower, indicating less inelastic scattering and fewer diffuse scattering when probing the clean Ru crystal. In both diffraction scans of h-BN and graphene the background increases by two orders of magnitude due to the increase of diffuse scattering. In addition, adlayers change the corrugation at the surface which is observed by the He atoms. X-ray studies showed that the peak-to-peak corrugation height of graphene is (0.82 ± 0.15), whereas for the uppermost Ru-atomic layer it is (0.19 ± 0.02).[11]

Performing a two-dimensional (2D) scan confirms that the diffraction peaks in the 1D angular diffraction scan of Figure 2(b) in the main text are correctly assigned to a (3×4) periodicity and cannot be explained as a subset of another periodicity or as domains with different rotations. Therefore we performed diffraction scans at various azimuthal orientations, since the BN_{II} structure has very distinct diffraction peaks in the high symmetry direction as well as along other azimuthal orientations. By rotation of the azimuthal angle of the sample a 2D-plot in reciprocal space can be created (see Supplementary Figure 5). The green cross marks the Ru diffraction peak while the red circles indicate the calculated positions of the (3×4) structure peaks. In the top panel three exemplary diffraction scans at specific azimuthal angles φ are depicted. Small angles close to the specular peak are not shown due to their high intensity in all scans. The identification of the peaks verifies the assumption that the (3×4) structure is present in addition to the h-BN layer on the surface and cannot be explained e.g. as being part of another superstructure or rotated domains of a (3×3) structure.

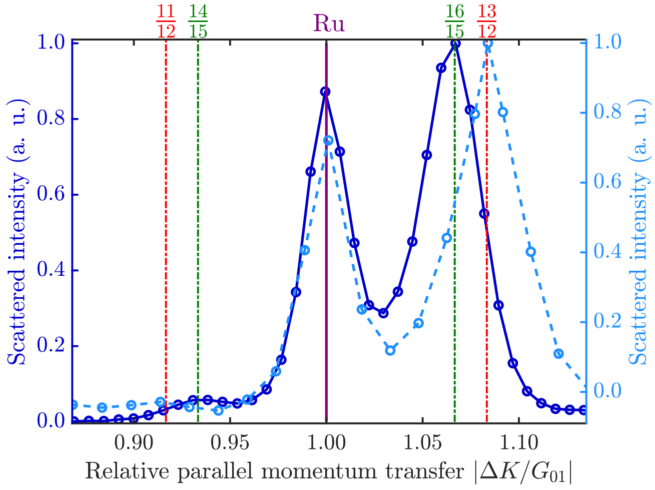

Supplementary Figure 6 Diffraction scans of the h-BN periodicity illustrate that the exact superstructure of the overlayer depends on the growth temperature, with the blue scan for h-BN grown at 1020 K and the dashed cyan curve for h-BN grown at 900 K. The h-BN peaks at the right-hand side of the Ru peak at $|\Delta K/G_{01}| = 1$ show, that h-BN adopts a larger superstructure with increasing growth temperature. Due to the decreasing lattice mismatch the overlayer adopts a 16/15 ratio versus a 13/12 ratio at 900 K. The “lock-in” effect (see text) is confirmed by the small (substrate) reconstruction peaks on the left-hand side. For better identification of the peaks a linear background was subtracted from the untreated data and the sample was subsequently cooled down to room temperature for the duration of the scan.

5 h-BN periodicity and reconstruction

The h-BN periodicity and superstructure are strongly dependent on the experimental parameters, in particular the growth temperature. It is well known that h-BN forms a Moiré pattern on

the Ru(0001) surface due to the small lattice mismatch between $a_{h-BN} = 2.505$ Å and $a_{Ru(0001)} = 2.706$ Å.[12,13] At room temperature, such a mismatch results in a superstructure where 13 unit cells of h-BN coincide with 12 unit cells of Ru: (13×13) on (12×12). On the other hand, previous studies on a similar substrate showed that the h-BN overlayer and the substrate lock in at the temperature during the growth with the strong interlayer bonding causing the superstructure ratio to remain constant after cooling back down.[73]

We show that the same holds for different growth temperatures of h-BN on Ru(0001). Detailed diffraction scans around the h-BN (01)-peak in Supplementary Figure 6 illustrate that for a h-BN synthesis at 1020 K (blue curve), the h-BN peak at $|\Delta K/G_{01}| = 1.067$ fits a superstructure ratio of 16/15 perfectly, as shown by the green vertical dash-dotted line. Upon growing the h-BN overlayer at a lower temperature of 900 K (cyan curve) the h-BN peak appears at a ratio of 13/12. The small peaks to the left of the first order Ru peak in Supplementary Figure 6 originate from the surface reconstruction with a 14/15 and 11/12 ratio, respectively. These reconstruction peaks can only arise if the system exhibits a true commensurate superstructure.[15,73]

Our HAS measurements show a strong temperature dependence and thus a strong "lock-in" effect, as further discussed below. Compared to X-ray diffraction where a commensurate 14-on-13 superstructure was reported,[29] we see that only h-BN growth at lower temperature (900 K with a borazine exposure of 15 L) followed by a slow subsequent cooling provides a 13 over 12 superstructure, similar to previous studies.[24] After all, compared to the h-BN/Rh(111) system,[18] the bonding strength of the N-atoms to the Ru substrate is predicted to increase and thus one expects a stronger "lock-in" effect on Ru as observed above. Moreover, due to HAS being strictly surface sensitive, our results can be interpreted as scattering that stems solely from the h-BN nanomesh while other methods may contain contributions from the substrate structure. E.g, a coincidental overlay of the flat h-BN monolayer on a completely flat Ru substrate would not give rise to a diffraction pattern as shown in Supplementary Figure 2(a) and Supplementary Figure 6. Together with the above reported additional structures, it confirms the complexity of the whole system and its dependence on minute changes of the growth parameters.

Looking at the thermal expansion coefficients of bulk h-BN and the Ru(0001) surface gives a rough estimation for the temperature at which the 13/12 superstructure is favourable. The thermal expansion of bulk h-BN[19] and the Ru surface[20] are given by:

$$a_{h-BN} = 2.505 - 7.42\times10^{-6}\cdot(T-297) + 4.79\times10^{-9}\cdot(T-297)^2 \quad (1a)$$

$$a_{Ru} = 2.706 + 9.22\times10^{-6}\cdot(T-293) \quad (1b)$$

Here the lattice constant for Ru $a_{Ru} = 2.706$ Å was taken for a surface temperature of 293 K, with 297 K for a_{h-BN}, hence the subtraction of these values.

The Ru thermal expansion is depicted in the upper left panel of Supplementary Figure 7, while the slope of bulk h-BN is shown as a blue line in the lower left panel. In addition, the thermal

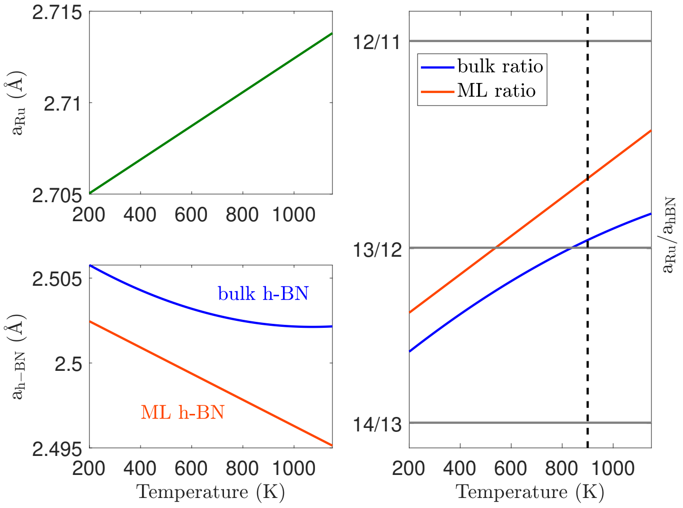

Supplementary Figure 7 Thermal expansion for the Ru surface (upper left panel) and bulk h-BN as well as monolayer (ML) h-BN (lower left panel). The ratio of the Ru surface lattice constant and the h-BN lattice constant versus temperature (right panel) provides an estimate of the expected superstructure. The grey horizontal lines depict the respective superstructure ratios while the dashed vertical line indicates a growth temperature of 900 K.

expansion for a single monolayer (ML) of h-BN as calculated by Thomas *et al.*[21] is drawn in orange. Taking the ratio of the values for h-BN and Ru then yields the expected superstructure at a given surface temperature, as shown in the right panel of Supplementary Figure 7. The expected fraction of 13/12 nicely fits the value of 900 K when using the bulk value of the thermal expansion.

As mentioned in the main text, HAS can also be used to determine the crystal quality and bonding to the substrate of the 2D material as e.g. shown for CVD-grown graphene[22–24] In particular inter-layer bonding of h-BN in comparison to graphene, possibly via phonon measurements would be an interesting route to pursue in future studies[24].

As also shown for layered materials, a measurement of the angular spread of the specular can be used to provide an estimate of the surface quality since the peak broadening is proportional to the average domain size, also known as the surface coherence length[25]. The same information can be obtained from the width of the diffraction peaks, although here the angular broadening is convoluted with the energy spread of the incident beam. Upon comparing the full width at half maximum (FWHM) of the h-BN peak in Supplementary Figure 6 at $(1.0\pm0.1)^\circ$ with the first order diffraction peak of pristine Ru in Supplementary Figure 4 (FWHM= $(0.68\pm0.03)^\circ$) we note that the crystalline quality of the complete h-BN layer is comparable to that of the pristine Ru(0001) surface.

6 Supplementary discussion

In the following we discuss further scenarios of the BN_{II} structure. As mentioned in the main text, the surface temperature strongly influences the kinetics and thus the duration and appearance of the additional superstructures. At temperatures above 1000 K

the (3×4) structure (BN_{II}) slowly vanishes (see Figure 5 in the main text) which leads to the assumption that either strongly bound atoms/molecules desorb into the gas phase or convert into another structure. As mentioned earlier the dehydrogenation of borazine already starts at lower temperatures[26] leading to the assumption that the adsorbed species on Ru(0001) are at least partly dehydrogenated.

In the following we provide several scenarios for the origin of the (3×4) structure and discuss their plausibilities. The results could be interpreted as if borazine converts upon adsorption to both h-BN and a (3×3) structure (BN_I). However, given the results which are reported in the main paper, it is clear that borazine only adsorbs in a (3×3) superstructure, and at 880 K a (relatively fast) conversion to h-BN occurs. The h-BN and BN_I structure grow together until the BN_I reservoir is depleted, and no more h-BN is created. At this point we can conclude that the (3×4) (BN_{II}) is not a precursor to h-BN and is also not converted from the BN_I structure. Since the (3×3) peaks degrade completely, the rise of the BN_{II} structure does not compete with the conversion of the BN_I structure to h-BN.

When looking at Figure 5 in the main paper one might also think that after the BN_I structure vanishes and the h-BN peak saturates, that the h-BN monolayer is complete and the additional borazine exposure gives rise to a second layer being formed. This layer could consist of partly dehydrogenated borazine forming a periodic structure on top of the existing h-BN layer. According to literature, the CVD process for h-BN growth is usually considered to be self-terminating after a single layer, while some works also showed that multilayers are formed,[27] however, typically these require different growth approaches.[28–31] As described in the main paper, from our experimental observations we can rule out the behaviour of multilayer h-BN growth and ascribed the BN_{II} structure to a second chemisorbed layer on top of h-BN.

Another possible scenario would be the growth of a superstructure in-between the already grown h-BN islands. As mentioned in the main manuscript an earlier work investigated the CVD growth of h-BN on Ir(111) and identified a (6×2) superstructure in-between the h-BN islands.[32] A similar behaviour could lead to the formation of a (3×4) structure in-between the h-BN islands on Ru. This intermediate structure eventually upon further borazine exposure converts into h-BN which connects the previously formed h-BN islands. However the areas which formed under this condition are less stable since they convert back to a (3×4) structure upon heating of the sample (see phenomenological cycle equation in the main paper). Upon further annealing of the surface the structures in-between the stable h-BN islands eventually desorb from the surface leaving behind some h-BN islands.

7 Outlook for other 2D materials

We hope that our initial findings encourage future investigations to give insight on the peculiar intermediate structures during the CVD growth of h-BN on Ru(0001). In fact we note that there is some preliminary experimental evidence as shown in Supplementary Figure 8, that an intermediate / precursor structure exists also for CVD of graphene on Ni(111) at temperatures below the best growth conditions. Only upon heating to 730 K the additional diffraction peak vansih, leaving just the (1×1) graphene/Ni(111) structure behind[33].

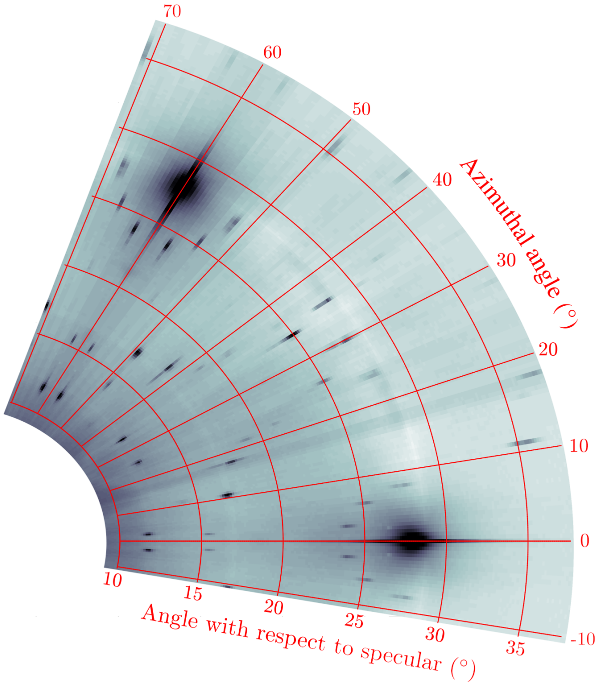

Supplementary Figure 8 A 2-dimensional diffraction scan of CVD-grown graphene on Ni(111) at temperatures below the “ideal” growth temperatures, reveals additional diffraction peaks which transform only upon heating to 730 K into the (1×1) graphene/Ni(111) structure.

Supplementary References

1 A. Tamtögl, E. A. Carter, D. J. Ward, N. Avidor, P. R. Kole, A. P. Jardine, J. Ellis and W. Allison, *Rev. Sci. Instrum.*, 2016, **87**, 066108.

2 S. J. Clark, M. D. Segall, C. J. Pickard, P. J. Hasnip, M. I. J. Probert, K. Refson and M. C. Payne, *Z. Kristallogr. Cryst. Mater.*, 2009, **220**, 567–570.

3 D. Vanderbilt, *Phys. Rev. B*, 1990, **41**, 7892–7895.

4 H. J. Monkhorst and J. D. Pack, *Phys. Rev. B*, 1976, **13**, 5188–5192.

5 J. P. Perdew, K. Burke and M. Ernzerhof, *Phys. Rev. Lett.*, 1996, **77**, 3865–3868.

6 A. Tkatchenko and M. Scheffler, *Phys. Rev. Lett.*, 2009, **102**, 073005.

7 F. Späth, J. Gebhardt, F. Düll, U. Bauer, P. Bachmann, C. Gleichweit, A. Görling, H.-P. Steinrück and C. Papp, *2D Mater.*, 2017, **4**, 035026.

8 B. Borca, S. Barja, M. Garnica, M. Minniti, A. Politano, J. M. Rodriguez-García, J. J. Hinarejos, D. Farías, A. L. V. d. Parga and R. Miranda, *New J. Phys.*, 2010, **12**, 093018.

9 D. Maccariello, D. Campi, A. Al Taleb, G. Benedek, D. Farías, M. Bernasconi and Miranda, *Carbon*, 2015, **93**, 1–10.

10 D. Martoccia, T. Brugger, M. Björck, C. Schlepütz, S. Pauli, T. Greber, B. Patterson and P. Willmott, *Surf. Sci.*, 2010, **604**, L16–L19.

11 D. Martoccia, M. Björck, C. M. Schlepütz, T. Brugger, S. A. Pauli, B. D. Patterson, T. Greber and P. R. Willmott, *New Journal of Physics*, 2010, **12**, 043028.

12 W. Paszkowicz, J. B. Pelka, M. Knapp, T. Szyszko and S. Podsiadlo, *Appl. Phys. A*, 2002, **75**, 431–435.

13 J. W. Arblaster, *Platinum Metals Review*, 2013, **57**, 127–136.

73 D. Martoccia, S. Pauli, T. Brugger, T. Greber, B. Patterson and P. Willmott, *Surf. Sci.*, 2010, **604**, L9–L11.

15 N. Leconte and J. Jung, *2D Mater.*, 2020, **7**, 031005.

29 D. Martoccia, T. Brugger, M. Björck, C. Schlepütz, S. Pauli, T. Greber, B. Patterson and P. Willmott, *Surf. Sci.*, 2010, **604**, L16–L19.

24 A. Goriachko, He, M. Knapp, H. Over, M. Corso, T. Brugger, S. Berner, J. Osterwalder and T. Greber, *Langmuir*, 2007, **23**, 2928–2931.

18 R. Laskowski, P. Blaha and K. Schwarz, *Phys. Rev. B*, 2008, **78**, 045409.

19 R. S. Pease, *Acta Crystallographica*, 1952, **5**, 356–361.

20 E. Ferrari, L. Galli, E. Miniussi, M. Morri, M. Panighel, M. Ricci, P. Lacovig, S. Lizzit and A. Baraldi, *Phys. Rev. B*, 2010, **82**, 195420–.

21 S. Thomas, K. M. Ajith, S. Chandra and M. C. Valsakumar, *J. Phys.: Condens. Matter*, 2015, **27**, 315302–.

22 G. Anemone, E. Climent-Pascual, H. K. Yu, A. Al Taleb, F. Jiménez-Villacorta, C. Prieto, A. M. Wodtke, A. De Andrés and D. Farías, *RSC Adv.*, 2016, **25**, 21235.

23 H. Shichibe, Y. Satake, K. Watanabe, A. Kinjyo, A. Kunihara, Y. Yamada, M. Sasaki, W. W. Hayes and J. R. Manson, *Phys. Rev. B*, 2015, **91**, 155403.

24 A. A. Taleb, G. Anemone, R. Miranda and D. Farías, *2D Mater.*, 2018, **5**, 045002.

25 A. Tamtögl, P. Kraus, N. Avidor, M. Bremholm, E. M. J. Hedegaard, B. B. Iversen, M. Bianchi, P. Hofmann, J. Ellis, W. Allison, G. Benedek and W. E. Ernst, *Phys. Rev. B*, 2017, **95**, 195401.

26 F. Orlando, R. Larciprete, P. Lacovig, I. Boscarato, A. Baraldi and S. Lizzit, *J. Phys. Chem. C*, 2012, **116**, 157–164.

27 P. R. Kidambi, R. Blume, J. Kling, J. B. Wagner, C. Baehtz, R. S. Weatherup, R. Schloegl, B. C. Bayer and S. Hofmann, *Chem. Mater.*, 2014, **26**, 6380–6392.

28 A. A. Tonkikh, E. N. Voloshina, P. Werner, H. Blumtritt, B. Senkovskiy, G. Güntherodt, S. S. P. Parkin and Y. S. Dedkov, *Sci. Rep.*, 2016, **6**, 23547.

29 A.-R. Jang, S. Hong, C. Hyun, S. I. Yoon, G. Kim, H. Y. Jeong, T. J. Shin, S. O. Park, K. Wong, S. K. Kwak, N. Park, K. Yu, E. Choi, A. Mishchenko, F. Withers, K. S. Novoselov, H. Lim and H. S. Shin, *Nano Lett.*, 2016, **16**, 3360–3366.

30 N. Guo, J. Wei, L. Fan, Y. Jia, D. Liang, H. Zhu, K. Wang and D. Wu, *Nanotechnology*, 2012, **23**, 415605.

31 P. Sutter, J. Lahiri, P. Zahl, B. Wang and E. Sutter, *Nano Lett.*, 2013, **13**, 276–281.

32 M. Petrović, U. Hagemann, M. Horn-von Hoegen and F.-J. Meyer zu Heringdorf, *Appl. Surf. Sci.*, 2017, **420**, 504–510.

33 A. Tamtögl, E. Bahn, J. Zhu, P. Fouquet, J. Ellis and W. Allison, *J. Phys. Chem. C*, 2015, **119**, 25983–25990.
